# Diphtheria

**Published:** 1875-04

**Authors:** 


DR. JACOBI, OF NEW YORK, ON DIPHTHERIA.— * * * He said the remedies which he uses are few in number and simple in character. * * * He looks after the mouth and the pharynx of the children among his acquaintances as a regular thing. Eruptions on the head must be removed, and glandular swellings around the neck prevented or speedily cured. The same may be said of nasal catarrh and catarrh of the pharynx. Hypertrophied tonsils must not be exsected at a time when diphtheria prevails, for at such a time every wound is apt to become diphtheritic, and hardly any operation inside the mouth will heal without becoming diphtheritic. For the same reason he postpones almost every sort of bloody operation during an epidemic of diphtheria, if possible. Lately he had seen the whole wound of an operation performed by one of the most prominent operators in the city, in a house where there was none of this disease before, become diphtheritic and jeopardize the success of the operation.

He spoke of the chlorate of potassium and the chloride of sodium as a means of preventing the disease. He did not, however, place much reliance on these agents as remedies in diphtheria, yet he gave chlorate of potassium in almost every case. * * He gives it for its effect on the inflamed mouth and pharynx, but not for the diphtheria. It acts as a preventive by restoring the mucous membrane to its normal condition. He does not do much more in tonsillar diphtheria. As this is a benign affection, it is of much greater importance to prevent it from spreading than to remove it from the tonsils, where its connections with the systems of the blood and lymphatics is so limited. In order to have the full effect, he insists upon frequent administration. Doses ought to follow each other in rapid succession: at least every hour, every half-hour, or every quarter of an hour a small dose ought to be given, to keep up a constant control over the endangered mucous membrane with the remedy. From a halfdrachm to a drachm may be given during the twenty-four hours. As many families are acquainted with the remedy and use it without being bidden, see to it that the dose is not too large. The death of Dr. Fountain, of Davenport, Iowa, and of many others, from over-doses of chlorate of potassium, ought to be heeded.

In mild cases of tonsillar diphtheria Dr. Jacobi sometimes tries to remove or destroy the membrane when the latter is accessible. He insists upon the latter being the case, because, in his opinion, the use of solid sticks or of the mineral acids has done more harm than good. Where he cannot reach the diphtheritic deposit and remove it thoroughly, usually with the concentrated carbolic acid, he lets it alone altogether. The experience is not new that abrasion of the mucous membrane and injury to the epithelium will spread this process in a very short time. In many of the simpler cases of tonsillar diphtheria, he gives chlorate of potassium combined with lime-water or tinctura ferri chloridi, ʒss. to ʒij. a day, and generally mixed with a little glycerin, for the purpose of keeping the remedy in longer contact with the diseased surface, if not for its own effects. * * *

Whatever is to be done in diphtheria must be done early. More than anything else, the doctor urges attention to feeding. Remembering the greediness of lymph-vessels when the chyle-vessels are not supplied, he feeds as well as the digestive powers of the patient will permit, always keeping in view, however, the fact that the stomach of a fever-patient must be carefully looked after. * * *

What are the dangers of nasal diphtheria? Rapid absorption, putrefaction, inhalation of vile smells. The indications are clear enough. The surface of the nose must be cleansed and disinfected. When you begin early, the layers of epithelium are less liable to be involved in the diphtheritic lesions. Your disinfectant will then be successful, and your injection will wash the surface clean. No strong disinfectant is required: two or four four grains of carbolic acid to the ounce of water is enough. Injections must be made into each nostril until the stream flows freely. This should be done every hour, or oftener if necessary. At the same time, care must be taken that none of the liquid reaches the fauces. Of otitis there is no fear. Probably the Eustachian tube is closed by catarrhal or diphtheritic swelling. He has never seen any difficulty arising from injections. A common syringe suffices; the nasal douche is much better. In neglected cases the syringe will open a closed-up nares. * * *

There is only one way to save a case of nasal diphtheria, and that is by disinfection and washing of the parts. What disinfectant is preferable? He avoided, in the first place, those which stain the surroundings, and those which coagulate. For that reason the various preparations of iron are not used, and also the permanganate of potassium. Carbolic acid has been frequently used by him. When there is no smell, lime-water has often been used, pure, or somewhat diluted, for its solvent effect. Internal disinfectants and antiseptics are of no effect. But, above all, attendance is necessary. He has not lost a single case in his own practice for years. He sees them early, or has them looked after, and takes care that his orders are strictly obeyed.—*Med. Times.*

